# Metabolic regulation of the neural stem cell fate: Unraveling new connections, establishing new concepts

**DOI:** 10.3389/fnins.2022.1009125

**Published:** 2022-10-21

**Authors:** Ioannis Angelopoulos, Georgios Gakis, Kyriakos Birmpas, Christina Kyrousi, Evagelia Eva Habeos, Konstantina Kaplani, Zoi Lygerou, Ioannis Habeos, Stavros Taraviras

**Affiliations:** ^1^Department of Physiology, Medical School, University of Patras, Patras, Greece; ^2^First Department of Psychiatry, Medical School, National and Kapodistrian University of Athens, Eginition Hospital, Athens, Greece; ^3^University Mental Health, Neurosciences and Precision Medicine Research Institute “Costas Stefanis”, Athens, Greece; ^4^Department of General Biology, School of Medicine, University of Patras, Patras, Greece; ^5^Division of Endocrinology, Department of Internal Medicine, University of Patras, Patras, Greece

**Keywords:** metabolism, neural stem cell niche, subventricular zone (SVZ), ependymal, neural stem cells, cell mechanics, ciliopathies

## Abstract

The neural stem cell niche is a key regulator participating in the maintenance, regeneration, and repair of the brain. Within the niche neural stem cells (NSC) generate new neurons throughout life, which is important for tissue homeostasis and brain function. NSCs are regulated by intrinsic and extrinsic factors with cellular metabolism being lately recognized as one of the most important ones, with evidence suggesting that it may serve as a common signal integrator to ensure mammalian brain homeostasis. The aim of this review is to summarize recent insights into how metabolism affects NSC fate decisions in adult neural stem cell niches, with occasional referencing of embryonic neural stem cells when it is deemed necessary. Specifically, we will highlight the implication of mitochondria as crucial regulators of NSC fate decisions and the relationship between metabolism and ependymal cells. The link between primary cilia dysfunction in the region of hypothalamus and metabolic diseases will be examined as well. Lastly, the involvement of metabolic pathways in ependymal cell ciliogenesis and physiology regulation will be discussed.

## Introduction

### Neural stem cell niche organization and physiology

**FIGURE 1:**
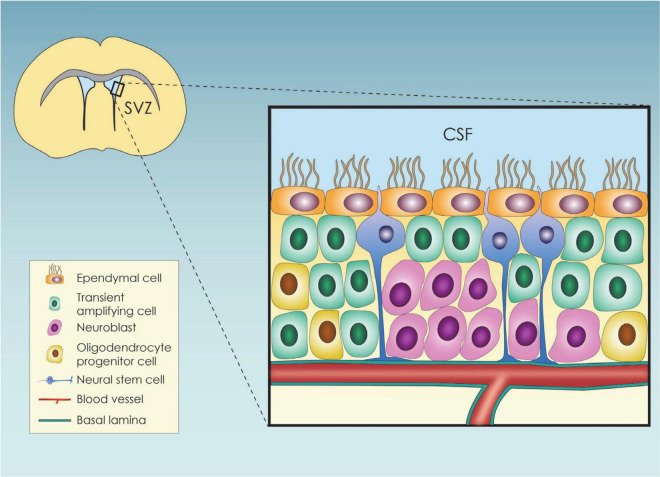
Schematic representation of the subventricular zone (SVZ) neural stem cell niche and its cellular components; SVZ, subventricular zone; CSF, cerebrospinal fluid.

The mammalian brain cortex originates from neuroepithelial stem cells (NESCS) or primary neural stem cells (pNSCs) (Solozobova et al., 2012), which differentiate toward radial glial cells (RGCs) during embryogenesis (Malatesta et al., 2008). RGCs, in turn, generate nascent projection neurons (Götz and Huttner, 2005; Molnár et al., 2019) that form a six-layered neocortex in an inside-out structural pattern, with the earlier migrating newborn neurons generating the deep cortical layers, and vice versa (Agirman et al., 2017). Toward the end of embryonic neurogenesis, the RGC scaffold detaches from the apical surface and leads to astrocyte and ependymal cell (EC) generation through asymmetric divisions (Molnár et al., 2019). RGCs also give rise to the adult neural stem cells (aNSCs) (Malatesta et al., 2008), which in the adult life are organized within specialized microenvironments, called neural stem cell niches (NSCN) (Andreotti et al., 2019). So far, two adult neurogenic areas have been established, the subgranular zone (SGZ) and the ventricular-subventricular zone (V-SVZ) niches, with the former being the main site of hippocampal neuronal generation and the latter being the main region from where the produced neuroblasts migrate toward the olfactory bulbs where they further differentiate into neurons (Altmann et al., 2019). NSCs at the SGZ are responsible for generating new excitatory neurons that colonize the dentate gyrus (DG) and contribute to adaptable memory development (Merkle et al., 2007). Within the niche, type 1 hippocampal stem cells are radial glia-like progenitors that divide at a low rate mainly asymmetrically to produce the type 2 cells, rapidly dividing (transient amplifying) progenitors. Type 2 cells initially have glial characteristics (also called Type-2a cells), but shortly after their generation change to express more neuronal characteristics, which are then designated as Type-2b cells (Kempermann et al., 2015). The progeny of type 2 cells differentiates into neuroblasts which shift their orientation from tangential to the length of the SGZ to a more polarized morphology to assume an essentially vertical orientation. Then, neuroblasts slowly maturate into granule cells, fully integrated into the hippocampal circuitry (Dantzer et al., 2008; Duan et al., 2008). Apart from intrinsic factors, it has been shown that extrinsic cues such as enriched odor exposure, aerobic exercise, increased stress, and dietary restriction can also regulate neurogenesis at the SGZ (Ming and Song, 2005). Within the V-SVZ, both proliferating and non-proliferating cells reside (Figure 1). The proliferating aNSCs residing in the region expressing an astroglial phenotype and give rise to astroglial-like cells which lack an apical contact with the cerebrospinal fluid (CSF) (Obernier and Alvarez-Buylla, 2019) and the so-called transient amplifying progenitor cells that can in turn lead to the production of neuroblasts (Obernier and Alvarez-Buylla, 2019). The non-proliferating cells are the ependymal cells (ECs) which are cuboidal to columnar ciliated cells (del Bigio, 1995), participating in various crucial physiological functions namely CSF circulation (Milošević et al., 2014) and trophic and metabolic support of the aNSCs (Murphy et al., 2017). They finetune adult neurogenesis through the so-called pinwheel structures surrounding the aNSCs cells (Paez-Gonzalez et al., 2011).

The specific cytoarchitecture of the V-SVZ niche is tightly associated with a specific and uniquely organized extracellular matrix (ECM) that regulates cell proliferation, differentiation, and migration throughout adulthood. The brain ECM alters its composition based on the developmental stage (Bandtlow and Zimmermann, 2000), but as far as its main structure is concerned, it comprises three main regions, the basal lamina the perineuronal nets and the neural interstitial matrix (Murphy et al., 2017). The brain ECM plays a fundamental role in neurogenesis, through NSC behavior regulation as it is a major source of chemical and biological cues, but it also provides physical and mechanical support to the NSCs all of which determine their fate (Shabani et al., 2021). Besides the classical SVZ and SGZ niches there are studies indicating that new neurons can be formed outside the SVZ and SGZ. The most intriguing non-canonical niche is the hypothalamic neurogenic site located in sub-ependymal zone of the third ventricle (HVZ). This zone consists of tanycytes, ciliated and non-ciliated ependymocytes which line the third ventricle as well as glial cells and neural cells (Rojczyk-Gołębiewska et al., 2014). The regulated hypothalamic neurogenesis in adult mice may play a previously unappreciated role in physiology and disease (Kokoeva et al., 2005; Rojczyk-Gołębiewska et al., 2014).

Identifying the mechanisms that govern pNSC and aNSC behavior is a multifactorial process which includes intrinsic and extrinsic mechanisms in the NSCN. The role of metabolism and specifically mitochondrial metabolism has been recently implicated as a regulator of NSC self-renewal and differentiation (O’Brien et al., 2015; Khacho et al., 2017; Knobloch and Jessberger, 2017). There is a reciprocal crosstalk between cell mechanics and metabolism (Romani et al., 2021). Given the anatomical complexity of the NSCN, and that EC borders with the CSF it is plausible that mechanical forces might affect SVZ homeostasis. The goal of this review is to provide an overview of the role of metabolism in the subventricular zone (SVZ) and subgranular zone (SGZ). The role of the hypothalamic niche will be briefly mentioned as it is linked to metabolic diseases (obesity). Our focus is the adult neural stem cell niche with occasional references to embryonic neural stem cells when it is deemed necessary. In terms of the cell types within the niche, the review mostly focuses on neural stem cells and multiciliated ependymal cells. The role of primary cilia in regulating the hypothalamic niche physiology is also being studied. The NSC fate is discussed in the context of different metabolic substrates and mitochondrial function. Additionally, the relationship between cerebrospinal fluid (CSF), ependymal cells and NSCN is also being studied in reference to metabolism. Lastly, although ECs have motile cilia, we have also included studies on both motile and non-motile cilia to gain a broader understanding of the issue (Satir and Christensen, 2007; Kishimoto and Sawamoto, 2012).

## Metabolic requirements of the neural stem cell niche

### Metabolic control of neural stem cell fate (self-renewal vs. differentiation)

As mentioned previously, neurogenesis is an important and intricate process carried on postnatally in distinct niches such as SVZ and SGZ (Bond et al., 2015). Understanding the NSC behavior is of paramount importance as it will unravel fundamental mechanisms occurring during neurogenesis (Taverna et al., 2014). In the recent years, the concept of metabolic reprogramming, which is the change in a cell’s metabolic activity, usually from glycolysis to oxidative phosphorylation (OXPHOS), depending on the current needs, is increasingly considered a fundamental factor regulating stem cell fate (Cliff and Dalton, 2017). Consequently, understanding what triggers metabolic reprogramming and how cell metabolism directs NSC fate decisions may provide new insight into the brain’s regenerative potential.

**FIGURE 2:**
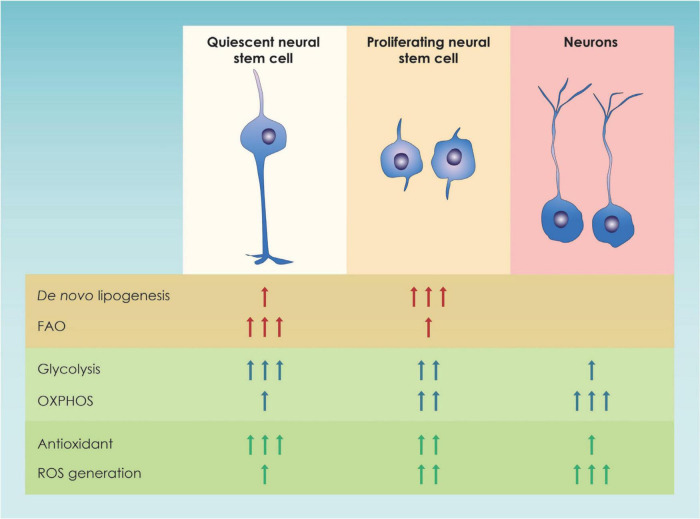
Graphic illustration of the main metabolic pathways of neural stem cells. In this figure the major changes in metabolic pathways occurring in quiescent and proliferating neural stem progenitor cells (NSPCs) as well as in immature neurons in the adult dentate gyrus and the developing forebrain are shown; FAO, Fatty Acid Oxidation; OXPHOS, Oxidative Phosphorylation; ROS, Reactive Oxygen Species.

Focusing on NSCs, cell metabolism has been shown to play a crucial role in a variety of processes, such as NSC proliferation, differentiation, and quiescence (Ito and Suda, 2014; Chandel et al., 2016) (Figure 2). In particular, NSC proliferation both during the embryonic and the adult period is linked to higher levels of glycolysis while, NSC differentiation is linked to an upregulation of genes involve in OXPHOS (Llorens-bobadilla et al., 2015; Agostini et al., 2016; Zheng et al., 2016; Maffezzini et al., 2020). This shift was once proposed to be a means to achieve a higher level of energy production but nowadays is considered a mechanism to drive NSC fate decisions (Homem et al., 2014; Stoll et al., 2015). This metabolic change from glycolysis to oxidative phosphorylation is especially essential during mid-embryogenesis, a time point at the peak of neurogenesis and neuronal production in cortical development (Mira and Morante, 2020) (Figure 2). Additional insights on the way glycolysis regulates NSC fate comes from studies suggesting that TP53 inducible glycolysis and apoptosis regulator (TIGAR), an endogenous inhibitor of glycolysis, is highly expressed in mature neurons and promotes NSC differentiation through acetyl coenzyme A (acetyl-CoA)-mediated histone acetylation (Zhou et al., 2019).

Lipid metabolism has also been found to be important in aNSC fate and in particular in hippocampal neurogenesis where it has been shown to control the proliferative activity of aNSCs. Fatty acid oxidase (FAO) seems to maintain NSC stemness whereas lipogenesis drives them toward differentiation (Maffezzini et al., 2020), a finding that has also been verified in humans (Namba et al., 2021). Specifically, fatty acid synthase (Fasn) is upregulated in NSCs whereas knockout Fasn mice showed decreased neurogenesis. The degree of lipogenesis is reduced in quiescence NSCs and is gradually increasing in proliferating NSCs, a process controlled by Spot14 through reduction of the amount of malonyl-CoA, the crucial substrate of Fasn to initiate lipogenesis (Knobloch et al., 2013). Spot14, a protein that has been previously shown to play a role in lipid metabolism (Zhu et al., 2001), is not expressed in aging NSC whereas it is induced by running leading to NSC proliferation by upregulating Fasn. This affected hippocampal-dependent learning and memory during neurogenesis that was shown to be tightly linked with exercise a process with high metabolic demands. The important role of Fasn in exercise-mediated cognitive enhancement was also highlighted in another study, implicating exercise-induced stimulation of neurogenesis (Chorna et al., 2013). In particular, human variant for the Fasn, R1812W, impaired adult hippocampal NSC activity, lipid accumulation in NSCs with ER stress and cognitive defects.

Neural stem cells (NSCs) in the SVZ producing enzymes required for fatty acid oxidation show constant increases in oxygen consumption upon treatment with a polyunsaturated fatty acid (Stoll et al., 2015). FAO inhibition, in turn, has been shown to result in a reduced NSC pool, which was due to increased differentiation and reduced self-renewal of NSCs, suggesting that FAO is indeed crucial for NSC maintenance (Xie et al., 2016). Knobloch et al. also demonstrated that the rate of FAO in NSCs in the hippocampal region regulated the balance between quiescent and proliferative states. Specifically, quiescent NSCs demonstrated high levels of carnitine palmitoyltransferase 1a (Cpt1a)-dependent FAO whereas is proliferative NSCs do not. Strikingly, manipulation of malonyl-CoA, favoring lipogenesis, is sufficient to induce quiescence exit and enhance NSC proliferation (Knobloch et al., 2017). Accumulation of lipids in the form of lipid droplets in the niche might influence NSC behavior. A study in Drosophila demonstrated that lipid droplets in the NSC niche sustained the proliferation of NSCs (neuroblasts) during starvation protecting both glia and neuroblasts by providing a ‘safe’ storage for polyunsaturated fatty acids during oxidative stress (Bailey et al., 2015). The above data from mouse model systems are reinforced by *in vitro* models of human brain development studies suggesting that altered lipid metabolism contributes to intellectual disability (Bowers et al., 2020) highlighting the importance of lipid accumulation in human neurogenesis shedding light on the beneficial role of the exercise and lipid metabolism in human behavior. Interestingly, lipid metabolism was also associated with human neurogenesis and cognitive deficits like intellectual disability and other neuropsychiatric disorders, with a characteristic example being the link between FAO deficits and autism (Xie et al., 2016).

From all the above, the importance of lipid metabolism was highlighted. Nevertheless, the energetic demands are different between actively proliferating and quiescent NSCs in the adult brain as specific metabolic processes were found different in the two categories of neural stem cells (Figure 2). In order to explain the lipid metabolism-associated metabolic switch, it has been speculated that proliferating NSCs activate lipogenesis in order to increase the amount of lipids available, that will later be use for plasma membrane synthesis (Knobloch, 2017). On the other hand, quiescent (non-proliferative) NSC use fatty acid oxidation to cover their energetic demands and maintain their stemness (van Noorden et al., 2022).

Glutaminolysis has also been shown to play a crucial role in NSC fate decisions. Specifically, glutaminolysis is required for the Rho GTPase Activating Protein 11B (ARHGAP11B)-mediated promotion of SVZ basal progenitors (BPs) proliferation during the embryonic life (Namba et al., 2020). What makes this finding even more interesting is that glutaminolysis is one of the few metabolic pathways which have been proven to regulate NSC fate decisions in humans and it appears to promote NSC proliferation (Namba et al., 2021).

Overall, there is evidence supporting the role of metabolism in the NSC fate and function regulation, reinforcing the notion that lipid and fatty acid metabolism contribute to human neurogenesis and behavior. It should also be noted that the transition from NSC to a neuronal lineage is accompanied by increased mitochondrial biogenesis, as well as downregulation of glycolysis and fatty acid oxidation pathways, reduction in glycolysis-related proteins, such as hexokinase 2 (HK2) and isoform A of lactate dehydrogenase (LDHA), switch from M2 isoform of the pyruvate kinase (PKM2) to its constitutively active M1 isoform (PKM1) and upregulation of OXPHOS-related genes (Zheng et al., 2016; Calvo-Garrido et al., 2019). Understanding how metabolism affects NSC behavior could potentially develop new therapeutic approaches to control it. In the next section the way mitochondria act as crucial regulators of NSC fate decisions by affecting metabolism will be discussed.

### Mitochondrial physiology, autophagy and epigenetics interplay: New players in neural stem cell fate regulation

**FIGURE 3:**
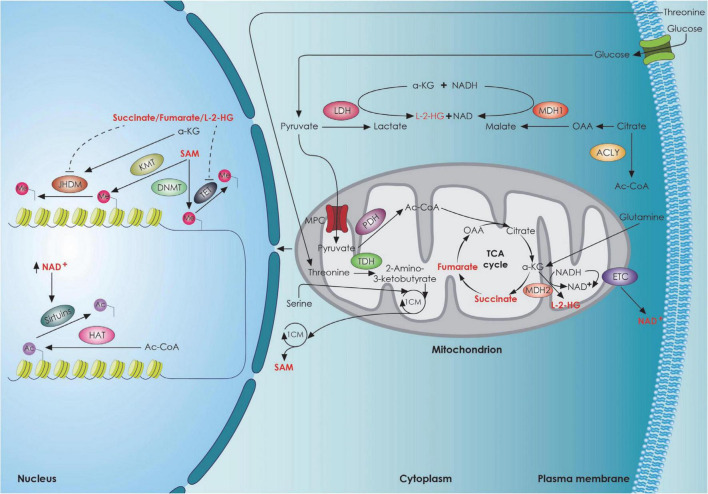
TCA cycle metabolites and stem cell fate. The interplay between the metabolic mitochondrial and epigenetic factors regulating neural stem cell fate decisions. 1CM, 1-carbon cycle; Ac, acetyl group; Ac-CoA, Acetyl-CoA; a-KG, Alpha-ketoglutarate; ACLY, ATP citrate lyase; DNMT, DNA methyl transferase; ETC, electron transport chain; HAT, histone acetyl transferase; JHDM, JmjC domain-containing histone demethylation protein; KMT, histone lysine N-methyltransferase; L-2-HG, L-2-hydroxyglutarate; LDH, lactate dehydrogenase; MDH1, malate dehydrogenase 1; MDH2, malate dehydrogenase 2; Me, Methyl group; MPC, mitochondrial pyruvate carrier; NAD, Nicotinamide adenine dinucleotide; OAA, oxaloacetate; PDH, pyruvate dehydrogenase; SAM, S-Adenosyl methionine; TDH, threonine dehydrogenase; TET, Ten Eleven Translocation enzyme.

Mitochondria are bioenergetic organelles involved in a variety of anabolic and catabolic functions (Chandel, 2021). Metabolites generated by the tricarboxylic acid (TCA) cycle and the electron transport chain, including adenosine triphosphate (ATP), reactive oxygen species (ROS), Acetyl-CoA, α-ketoglutarate (a-KG), and nicotinamide adenine dinucleotide (NAD)/NADH ratio, serve as signaling molecules regulating several aspects of stem cell function (Martínez-reyes and Chandel, 2020). Stem cells, including NSCs have several antioxidant defense systems regulated by transcription factors [Forkhead/winged helix box gene, group O (FoxO), Nuclear factor erythroid 2-related factor 2 (Nrf2)] to keep ROS within a narrow range. The low concentration of ROS is considered a hallmark of adult stem cells and plays an essential role in their homeostasis (Tsatmali et al., 2005; Go, 2012; Ma et al., 2015; Chandel et al., 2016; Shyh-Chang and Ng, 2017), ensuring their protection against oxidative damage. There is also evidence suggesting that mitochondrial ROS act as a rheostat to affect gene expression and regulate cell fate (Le Belle et al., 2011; Walton et al., 2012; Maryanovich and Gross, 2013). In particular, the Forkhead Box O-3 (Foxo3)-dependent antioxidant system is essential for the self-renewal of NSCs by asymmetric divisions (Renault et al., 2009; Webb et al., 2013). ROS-mediated NSC fate decisions are also relevant in aging. In a mouse model of advanced aging accumulating mitochondrial DNA (mtDNA) mutations with age [DNA Polymerase gamma A (PolgA) mutant mice], there is a decrease in quiescent NSCs in the adult SVZ accompanied by a loss of self-renewal that could be rescued with antioxidants was observed (Go, 2012). However, high ROS levels in NSCs can impair adult neurogenesis or shift NSC fate decisions toward the astroglial lineage at the expense of newborn neurons (Fishman et al., 2009; Ali et al., 2015). Mitochondrial dynamics, as well as mitochondrial crosstalk with other organelles (Yambire et al., 2020) play an important role in determining cell fate and function as well. Khacho and Slack (2018). The importance of mitochondria for stem cell function is supported by several studies demonstrating that mitochondrial dysfunction can lead to deleterious effects on stem cell function, both during development and in the adult brain (Khacho et al., 2016, 2017; Beckervordersandforth, 2017; Beckervordersandforth et al., 2017; Bahat and Gross, 2019; Chen et al., 2020). Several studies suggest that alterations in mitochondrial respiration (Figure 3) can impair adult neurogenesis (Calingasan et al., 2008; Beckervordersandforth et al., 2017; Khacho et al., 2017). This has been demonstrated using conditional deletion of genes integral to mitochondrial function and examining the effect on NSCs in the adult mouse. The genes that have been tested are the TFAM (transcription factor A, mitochondrial) (Beckervordersandforth et al., 2017), the α-KG-dehydrogenase complex (Calingasan et al., 2008) the mitochondrial oxidoreductase protein apoptosis initiating factor (AIF) (Khacho et al., 2017). Apart from the essential role that mitochondria play in adult NSCs, recent studies have also revealed the importance of mitochondrial dynamics during neural development, particularly during NSC fate decisions and neuronal differentiation. In addition to metabolic changes, mitochondria within NSCs undergo morphological changes as cells commit to a neuronal lineage in SVZ. These changes in shape are not consequential but rather direct the metabolic changes and regulate the fate of NSCs by mitochondrial-to-nuclear retrograde signaling mediated by ROS and transcription factor Nrf2 (Steib et al., 2014; Khacho et al., 2016). On the other hand, loss of dynamin-related protein 1 (DRP1) in uncommitted NSCs causes mitochondrial elongation, decreased mitochondrial ROS levels, and promotes NSC self-renewal capacity (Khacho et al., 2016). In support of the impact of morphological changes of mitochondria in neuronal commitment, Iwata et al. recently proposed that fusion vs. fission of mitochondria in ventricular zone NSCs during M-phase modulate neuronal commitment and differentiation (Iwata et al., 2020). It is important to note that the role of mitochondrial dynamics in NSC fate is mediated at the nuclear level, by means of mitochondrial to nuclear retrograde signaling, to regulate the transcription of self-renewal versus differentiation genes (Figure 3). Another important aspect in NSCs fate commitment is autophagy given that autophagy is lower in quiescent NSCs and promotes NSC activation and differentiation (Casares-Crespo et al., 2018). Mutations in autophagy related 5 (ATG5) and Autophagy and Beclin 1 Regulator 1 (Ambra1) components of autophagic machinery in NSCs or pharmacologic interventions that suppress autophagy impair early steps of stem and progenitor cells differentiation (Vázquez et al., 2012; Wang et al., 2013). It has also been shown that bi-allelic null mutations in p62 present with early-onset neurodegenerative disorders (Haack et al., 2016; Muto et al., 2018). Specifically, it resulted in a dramatic increase of lactate dehydrogenase A (LDHA) expression, which correlated with deficient neurodifferentiation due to the inability to upregulate genes important for OXPHOS (Calvo-Garrido et al., 2019), a phenotype rescuable by N-acetylcysteine, suggesting a role of p62 in oxygen sensing or ROS management and implying the role of autophagy in brain-related disorders (Lee et al., 2013; Deng et al., 2021). It is known that under oxidative stress p62 stabilizes Nrf2 (Jain et al., 2010; Komatsu et al., 2010). Deletion of p62 also rescued the NSC pool in the SVZ and dental gyrus of autophagy deficient FIP 200-KO mice, demonstrating an important role for p62 in neuronal development regulation, probably by regulating intracellular superoxide levels (Wang et al., 2016).

Epigenetic modifications, including DNA and histone modifications have also been shown to play a crucial role in the regulation of NSC fate (Yao et al., 2016) with histone demethylation and acetylation being the main modifications promoting NSC proliferation and self-renewal and methyl-CpG binding protein 2 (MECP2)-mediated histone methylation promoting terminal neuronal differentiation and synaptogenesis (Niklison-Chirou et al., 2020). Histone methylation has been shown to influence Notch1 (Zhang et al., 2015) and Huntingtin expression (Irmak et al., 2018; Lu et al., 2020; Alcalá-Vida et al., 2021). Histone acetylases and deacetylase also regulate proper NSC function through huntingtin expression regulation (Yildirim et al., 2019). Given the fact that NSC fate is both metabolically and epigenetically regulated, a link between metabolic and epigenetic regulation has been described (Figure 3). Specifically, it appears that specific metabolites promote a NSC epigenetic reprogramming, thus affecting NSC self-renewal and differentiation (Fawal and Davy, 2018). It has been shown that lipid-derived acetyl-CoA promotes histone hyperacetylation (McDonnell et al., 2016), a process involved in TIGAR-mediated NSC differentiation (Zhou et al., 2019). Flavin Adenine Dinucleotide (FAD) enhanced neuronal differentiation of human NSCs by facilitating nuclear localization of Lysine-specific demethylase 1 (LSD1) (Hirano and Namihira, 2016). In the epigenetic control of NSC fate, the mitochondria are critical regulators of this process through the cellular NAD^+^/NADH ratio regulation that can dictate the fate of stem cells *via* sirtuins (Imai and Guarente, 2014; Cai et al., 2016) (Figure 3). Sirtuin 1 (SIRT1) has been shown to be expressed mainly in stem cells, with a much lower expression during differentiation and this has been shown to be required for the maintenance and differentiation of stem cells (Calvanese et al., 2010). An increase in NAD^+^/NADH ratio inhibits adult hippocampal NSC self-renewal and promotes embryonic NSC neuronal differentiation (Ma et al., 2014; Zhang et al., 2016). Additional studies highlight that NAD^+^ is a key regulator of stem cell fate during the normal aging process. NAD-dependent Sirt1 activation contributes to quiescence in adult NSCs through peroxisome proliferator-activated receptor-gamma coactivator 1-alpha (PGC-1a) activation (Rodgers et al., 2005; O’Brien et al., 2015) and Forkhead Box O1 (FOXO1) deacetylation (Brunet et al., 2004; Renault et al., 2009; Webb et al., 2013). There are also studies demonstrating that genetic or pharmacological inhibition of Sirt1 in adult NSCs favors their exit from quiescence and stimulates their proliferation and differentiation (Rafalski et al., 2013; Saharan et al., 2013; Hu et al., 2014). It is worth noting that nicotinamide phosphoribosyl transferase (NAMPT), which partially controls NAD^+^ levels, declines with age in the mouse hippocampus (Stein and Imai, 2012). Deletion of NAMPT in adult NSCs and progenitor cells reduces their ability to self-renew, proliferate, and generate oligodendrocytes, which can be rescued by nicotinamide mononucleotide (NMN; a precursor of NAD^+^) supplementation (Stein and Imai, 2014). On the other hand, other studies have suggested that NAD-activated Sirt1 suppresses the quiescence and promotes proliferation and differentiation (Hisahara et al., 2008; Ma et al., 2014). However, activation of SIRT1 by resveratrol to mimic early neural developmental stress increased neural tube defects in mouse embryos (Li et al., 2017). Thus, tight regulation of NAD^+^/NADH ratio is essential for neurogenesis (Figure 3). Besides Sirt1, sirtuin 6 (SIRT6) might regulate cell fate during adult hippocampal neurogenesis (Okun et al., 2017).

### Primary cilia and neurogenesis: The hypothalamus paradigm

As it was mentioned in the introduction, RGCs can differentiate into aNSCs and ECs with the latter ones being multiciliated. RGCs, as most cells, have primary cilia to detect the environment and respond appropriately (Higginbotham et al., 2013). Primary cilia detect extracellular cues, such as mechanical flow and chemicals, and transduce these signals to regulate various processes, including differentiation, cell cycle regulation, neurotransmission, and metabolism (Berbari et al., 2009). In addition, many cells with primary cilia differentiate toward multiciliated ones, as is the case for postnatal radial glial cells in the SVZ which differentiate into multiciliated ependyma cells (Delgehyr et al., 2015).

Primary cilia themselves have been shown to be metabolically regulated with cholesterol metabolism being a key regulator of primary cilium physiology. One characteristic example is sterol regulatory element-binding protein 1 (SREBP1c), an important transcription factor for fatty acid and cholesterol biosynthesis, whose aberrant activation has been shown to suppress ciliary formation (Gijs et al., 2015). Dysregulated cholesterol metabolism also negatively affects ciliogenesis, as atorvastatin-mediated cholesterol depletion leads to reduced ciliary signaling, ciliation frequency and ciliary length (Maerz et al., 2019), which could explain the teratogenic effects of statins (Maerz et al., 2019). The above is linked to studies suggesting that atorvastatin promotes Notch1 expression and increase aNSC proliferation after stroke (Chen et al., 2008). In addition, proper cholesterol distribution among the different organelles is crucial for proper ciliogenesis (Maharjan et al., 2020).

Glucose metabolism also plays a significant role in primary cilium function, as glucose deprivation induces primary cilia formation through mammalian target of rapamycin complex 1 (mTORC1) inactivation and p27 activation (Takahashi et al., 2018). Additionally, methylglyoxal, a glycolytic intermediate metabolite, modulates Notch signaling to regulate neural progenitor cell (NPC) fate decision (Rodrigues et al., 2020).

As previously stated, the role of primary cilia is important in determining aNSC (Amador-Arjona et al., 2011; Khatri et al., 2014; Tong et al., 2014) and EC fate (Mirzadeh et al., 2010). The most characteristic paradigm of the above is the hypothalamic neurogenic niche, which comprises of two main layers, the hypothalamic ventricular zone (HVZ) and the hypothalamic proliferating zone (HPZ) (Rojczyk-Gołębiewska et al., 2014). In this niche, four types of radial glia-like tanycytes along with ciliated and non- ciliated ependymal cells are found (Rojczyk-Gołębiewska et al., 2014). Recent studies have demonstrated that tanycytes’ primary cilia are considered key players in hypothalamic neurogenesis (Rojczyk-Gołębiewska et al., 2014). Specifically, in embryonic hypothalamic niche primary cilia function determines hypothalamic neurogenesis. Primary cilia mediate early life programming of adiposity through lysosomal regulation in the developing mouse hypothalamus (Lee et al., 2020; Yamakawa et al., 2021). Primary cilia modulate energy homeostasis through centrally mediated control of feeding (hypothalamus) and peripheral tissue signaling and homeostasis (pancreas, skeletal muscle, adipocyte differentiation) (Rojczyk-Gołębiewska et al., 2014). Several pediatric obesity syndromes have been found to have mutations in genes related to primary cilia function such as Bardet-Biedl syndrome (BBS), Ahlstrom syndrome, Mental retardation, truncal obesity, retinal dystrophy and micropenis syndrome (MORM) and Carpenter syndrome (Ansley et al., 2003; Hampshire et al., 2006; Alessandri et al., 2010). Besides this, there are several studies suggesting the fundamental role of primary cilia in appetite regulation by leptin signaling. Several key molecules involved in appetite and satiety regulation such as leptin receptor, melanocortin-4-receptor (MC4R), or adenylate cyclase 3 are affected by the function of primary cilium (Ou et al., 2009; Han et al., 2014; Oh et al., 2015; Krashes et al., 2016; Grarup et al., 2018). Preadipocytes possess a primary cilium during differentiation that plays a critical role in their ability to become adipocytes (Marion et al., 2008; Kopinke et al., 2017). It is worth mentioning that primary cilia in human embryonic stem cells affect their commitment to neuroectoderm through the autophagy-Nrf2 axis (Jang et al., 2016).

### Ependymal maturation and their metabolic function in neural stem cell niches

The SVZ is sandwiched between a basement membrane on the one end (attached to blood capillaries) and ECs on the other end (del Bigio, 2010). EC have motile cilia (Delgehyr et al., 2015) and are exposed to the CSF mechanical forces (Siyahhan et al., 2014; Ringers et al., 2020). It has been already demonstrated that mechanical forces, especially shear stress, affect ciliogenesis (Sheng et al., 2020) and potentially might affect the stem cell niche. For the homeostasis of CSF flow to be achieved, ependymal cells need to maintain ciliary and planar cell polarity during and after ciliogenesis as it has been shown by Yamada et al. in hydrocephalus models, with an increase in CSF oscillation directly impedes normal cilia beating (Yamada et al., 2021).

Studies in renal epithelial cells have shown that primary cilia detect shear stress and stimulate lipophagy, promote mitochondrial biogenesis and increases ATP production which provides the energy required for metabolic reprogramming and cellular adaptation (Miceli et al., 2020). Similarly, ependymal cells have the potential to respond to CSF shear stress. In addition, although ependymal maturation is mainly a gene-controlled process, late findings have shown EC ciliogenesis is metabolically regulated, which comes in accordance with studies suggesting that metabolic dysregulation is involved in hydrocephalus pathophysiology (Caner et al., 1993; Kondziella et al., 2008; Lummis et al., 2019) and that ECs secrete a variety of metabolic products, such as apolipoprotein E (APOE) (Lee et al., 2012).

**FIGURE 4:**
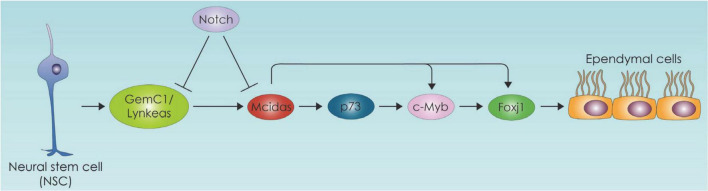
Neural stem cell differentiation toward the ependymal cell lineage pathway. The ependymal cell differentiation process is depicted which is primarily controlled by the Geminin family protein members. GemC1:Geminin coiled-coil domain-containing protein 1, Mcidas: Multiciliate Differentiation and DNA Synthesis Associated Cell Cycle Protein, Foxj1: Forkhead Box J1.

Ependymal cells (EC) act as a barrier as well as selective transporter of molecules such as fatty acids which enter ependymal cells and incorporated into triacyl glycerol compounds. This sequestration of fatty acids in the form of triacyl glycerol seem to be part of an active transport mechanism of essential fatty acids from CSF to central nervous system (CNS) (Etschmaier et al., 2011). On the other hand, lipids secreted from glial cells pass through the ependymal barrier into the CSF (Marin et al., 2013). EC play an important role regulating the biology of NSC within SVZ niche, by providing signals from CSF that influence adult NSC quiescence and proliferation (Mirzadeh et al., 2008; Tavazoie et al., 2008). Specifically, they secrete Noggin and thus favor neurogenesis by suppressing bone morphogenetic protein (BMP) (Lim et al., 2000; Lee et al., 2012; Yousef et al., 2015). Importantly within the SVZ and the subgranular zone adult NSCs require fatty acid oxidation for their proliferation and differentiation (Chorna et al., 2013; Knobloch et al., 2013; Stoll et al., 2015). With aging the SVZ ependymal cell number decreases, and these cells accumulate large lipid droplets which are vital for normal ependymal function (Bouab et al., 2011). Hamilton et al. have demonstrated in an Alzheimer’s mouse model that excess of lipid droplets in the ependymal lining of the VZ leads to decreased NSC proliferation suggesting that perturbed lipid metabolism in disease might be directly influencing NSC behavior (Hamilton et al., 2015). In another mouse model of Huntington’s disease (HD) lower 24S-hydroxycholesterol levels have been linked to increased neurodegeneration (Kacher et al., 2019). Lipidomic profiling of HD patients also stress the significant role of lipids which leads to a disrupted EC layer in the SVZ (Hunter et al., 2018). Additional evidence of the role of lipid metabolism in ependymal cells comes from studies suggesting that lipid-laden ECs are also linked to an increase in quiescent NSCs and neuroinflammation markers (Shimabukuro et al., 2016). One important aspect of the SVZ adult neurogenic niche is the regulation of NSC biology by ependymal cells (ECs). In this section we will focus on EC differentiation, which includes the process of EC ciliogenesis, and the metabolic regulation of these processes. Multiciliated ECs, are postmitotic cells derived from RGCs. They are committed toward the ependymal lineage in the mid embryogenesis and their generation is primarily controlled by the Geminin family proteins (Kyrousi et al., 2015, 2017; Lalioti et al., 2019b; Ortiz-Álvarez et al., 2019). We have previously shown that Geminin coiled-coil domain-containing protein 1 (GemC1) and Multiciliate Differentiation and DNA Synthesis Associated Cell Cycle Protein (Mcidas) operating in a hierarchical mode and transcriptionally activate Forkhead Box J1 (Foxj1), c-Myb and p73 (Kyrousi et al., 2015; Lalioti et al., 2019a) in EC differentiation (Figure 4).

In the study of Lalioti et al. deletion of GemC1 in mice triggered cell fate changes of neural progenitor cells of the brain into adult NSCs instead of the brain ependymal cells. These cell fate alterations were followed by global changes in chromatin accessibility of several genes promoters resulting in higher accessibility of NSCs-related promoters. Among others fatty acid binding protein 7 (Fabp7) and lactate dehydrogenase B (Ldhb) gene promoters which are important for fatty acid metabolism and lipid biosynthesis were found to display increased accessibility in GemC1-knockout mice (Lalioti et al., 2019b). The link between EC ciliogenesis and metabolism is also evident in the case of p73, which is the master transcriptional regulator of ciliogenesis (Jackson and Attardi, 2016) and has also been found to affect metabolism (Nemajerova et al., 2018). There is an intriguing possibility that p73 affects ciliogenesis through lipid metabolism. Specifically, p73 regulates cellular metabolism and energy production, directly through transcriptional regulation of several metabolic enzymes such as glutaminase 2 and glucose 6 phosphate dehydrogenase (Du et al., 2013). It also promotes glycolysis, amino acid uptake and biosynthesis of acetyl-CoA (Amelio et al., 2014). It is important to emphasize that p73 interacting with p63 promotes fatty acid oxidation and inhibits fatty acid synthesis (Sabbisetti et al., 2009; Su et al., 2012). The mechanism by which p73 affects fatty acid metabolism can be mediated by autophagy as indicated by studies in hepatocytes (He et al., 2013).

## Discussion and future directions

Here, we comprehensively reviewed the existing literature on Neural stem cell Niche regarding the role of metabolism in NSC fate with special emphasis in ciliogenesis. Over the years, significant progress has been made to unravel the mechanisms by which NSCs are differentiated toward neurons during embryonic and adult neurogenesis. Recently it has been discovered that cellular metabolism plays an important role in maintaining quiescent or proliferative NSCs and in modulating self-renewal and differentiation of NSCs. The main goal for future research is to unravel the metabolic adaptations that occur in NSCs that maintain their stemness or initiate their differentiation. Cell metabolism may serve as a signal integrator that “translates” a variety of signals into an integrated metabolic response that may affect cells at multiple levels, e.g., energy status, fuel utilization source, oxidative stress, and epigenetics. Thus, studying the interplay between transcriptional programs, morphogenic signaling, and its down-stream or up-stream regulation of the metabolic state may substantially improve our understanding of how NSPCs orchestrate the construction of the brain. Given that cilia, primary or motile, are in the interface between extracellular and intracellular space, their contribution in metabolic processes is not unexpected as discussed here. Although we do not fully understand the mechanisms of metabolic control of NSPC behavior yet, there is strong evidence that the metabolic state is a novel key player regulating the balance between stem cell quiescence/activation and subsequent differentiation. Identification and subsequent targeting of the compensatory metabolic pathways create opportunities for more efficient therapeutic strategies. Forward and unbiased metabolomics approaches should facilitate understanding this metabolic rewiring. Developing further knowledge on other aspects of neural stem cell metabolism such as metabolic compartmentalization, metabolic crosstalk with other cell types, and EC subtype-specific metabolic traits is another future challenge in this young field and a prerequisite to the development of novel therapeutic strategies.

## Author contributions

IA conceived the idea of this review and wrote together with GG, KB, and IH the first draft of this manuscript. CK, EH, KK, ZL, and ST supervised the writing of the manuscript, helped with writing, and provided their specialized insights based on their different backgrounds. IA produced all the schematic illustrations for the figures. All authors contributed to the article and approved the submitted version.

## References

[B1] Agirman G. Broix L. Nguyen L. (2017). Cerebral cortex development: An outside-in perspective. *FEBS Lett.* 591 3978–3992. 10.1002/1873-3468.12924 29194577

[B2] Agostini M. Romeo F. Inoue S. Niklison-Chirou M. V. Elia A. J. Dinsdale D. (2016). Metabolic reprogramming during neuronal differentiation. *Cell Death Differ.* 23 1502–1514. 10.1038/cdd.2016.36 27058317 PMC5072427

[B3] Alcalá-Vida R. Seguin J. Lotz C. Molitor A. M. Irastorza-Azcarate I. Awada A. (2021). Age-related and disease locus-specific mechanisms contribute to early remodelling of chromatin structure in Huntington’s disease mice. *Nat. Commun.* 12:364. 10.1038/s41467-020-20605-2 33441541 PMC7807045

[B4] Alessandri J. L. Dagoneau N. Laville J. M. Baruteau J. Hébert J. C. Cormier-Daire V. (2010). RAB23 mutation in a large family from Comoros Islands with carpenter syndrome. *Am. J. Med. Genet. A* 152 982–986. 10.1002/ajmg.a.33327 20358613

[B5] Ali A. A. H. Schwarz-Herzke B. Stahr A. Prozorovski T. Aktas O. von Gall C. (2015). Premature aging of the hippocampal neurogenic niche in adult Bmal1-deficient mice. *Aging* 7 435–449. 10.18632/aging.100764 26142744 PMC4505169

[B6] Altmann C. Keller S. Schmidt M. H. H. (2019). The role of SVZ stem cells in Glioblastoma. *Cancers* 11:448. 10.3390/cancers11040448 30934929 PMC6521108

[B7] Amador-Arjona A. Elliott J. Miller A. Ginbey A. Pazour G. J. Enikolopov G. (2011). Primary cilia regulate proliferation of amplifying progenitors in Adult Hippocampus: Implications for learning and memory. *J. Neurosci.* 31 9933–9944. 10.1523/JNEUROSCI.1062-11.2011 21734285 PMC3758574

[B8] Amelio I. Antonov A. A. Catani M. V. Massoud R. Bernassola F. Knight R. A. (2014). TAp73 promotes anabolism. *Oncotarget* 5 12820–12934. 10.18632/oncotarget.2667 25514460 PMC4350352

[B9] Andreotti J. P. Silva W. N. Costa A. C. Picoli C. C. Bitencourt F. C. O. Coimbra-campos L. M. C. (2019). Seminars in cell & developmental biology neural stem cell niche heterogeneity. *Semin. Cell Dev. Biol.* 95 42–53. 10.1016/j.semcdb.2019.01.005 30639325 PMC6710163

[B10] Ansley S. J. Badano J. L. Blacque O. E. Hill J. Hoskins B. E. Leitch C. C. (2003). Basal body dysfunction is a likely cause of pleiotropic BardetBiedl syndrome. *Nature* 425 628–633. 10.1038/nature02030 14520415

[B11] Bahat A. Gross A. (2019). cro Mitochondrial plasticity in cell fate regulation. *J. Biol. Chem.* 294 13852–13863. 10.1074/jbc.REV118.000828 31383739 PMC6755789

[B12] Bailey A. P. Koster G. Guillermier C. Lechene C. P. Postle A. D. Gould A. P. (2015). antioxidant role for lipid droplets in a stem cell niche of *Drosophila* article antioxidant role for Lipid droplets in a stem cell niche of *Drosophila*. *Cell* 163 340–353. 10.1016/j.cell.2015.09.020 26451484 PMC4601084

[B13] Bandtlow C. E. Zimmermann D. R. (2000). Proteoglycans in the developing brain: New conceptual insights for old proteins. *Physiol. Rev.* 80 1267–1290. 10.1152/physrev.2000.80.4.1267 11015614

[B14] Beckervordersandforth R. (2017). Mitochondrial metabolism-mediated regulation of Adult neurogenesis. *Brain Plast.* 3 73–87. 10.3233/BPL-170044 29765861 PMC5928529

[B15] Beckervordersandforth R. Ebert B. Schäffner I. Moss J. Fiebig C. Shin J. (2017). Role of mitochondrial metabolism in the control of early lineage progression and aging phenotypes in Adult Hippocampal neurogenesis. *Neuron* 93 560–573e6. 10.1016/j.neuron.2016.12.017 28111078 PMC5300896

[B16] Berbari N. F. O’Connor A. K. Haycraft C. J. Yoder B. K. (2009). The primary cilium as a complex signaling center. *Curr. Biol.* 19:R526–R535. 10.1016/j.cub.2009.05.025 19602418 PMC2814769

[B17] Bond A. M. Ming G. L. Song H. (2015). Adult *Mammalian* neural stem cells and neurogenesis: Five decades later. *Cell Stem Cell* 17 385–395. 10.1016/j.stem.2015.09.003 26431181 PMC4683085

[B18] Bouab M. Paliouras G. N. Aumont A. Forest-Bérard K. Fernandes K. J. L. (2011). Aging of the subventricular zone neural stem cell niche: Evidence for quiescence-associated changes between early and mid-adulthood. *Neuroscience* 173 135–149. 10.1016/j.neuroscience.2010.11.032 21094223

[B19] Bowers M. Liang T. Gonzalez-bohorquez D. Jessberger S. Bowers M. Liang T. (2020). FASN-Dependent lipid metabolism links neurogenic stem/progenitor cell activity to learning and memory deficits. *Cell Stem Cell* 27 98–109.e11. 10.1016/j.stem.2020.04.002 32386572

[B20] Brunet A. Sweeney L. B. Sturgill J. F. Chua K. F. Greer P. L. Lin Y. (2004). Stress-dependent regulation of FOXO transcription factors by the SIRT1 deacetylase. *Science* 303 2011–2015. 10.1126/science.1094637 14976264

[B21] Cai Y. Xu L. Xu H. Fan X. (2016). SIRT1 and neural cell fate determination. *Mol. Neurobiol.* 53 2815–2825. 10.1007/s12035-015-9158-6 25850787

[B22] Calingasan N. Y. Ho D. J. Wille E. J. Campagna M. V. Ruan J. Dumont M. (2008). Influence of mitochondrial enzyme deficiency on adult neurogenesis in mouse models of neurodegenerative diseases. *Neuroscience* 153 986–996. 10.1016/j.neuroscience.2008.02.071 18423880 PMC2907648

[B23] Calvanese V. Lara E. Suárez-Álvarez B. Dawud R. A. Vázquez-Chantada M. Martínez-Chantar M. L. (2010). Sirtuin 1 regulation of developmental genes during differentiation of stem cells. *Proc. Natl. Acad. Sci. U.S.A.* 107 13736–13741. 10.1073/pnas.1001399107 20631301 PMC2922228

[B24] Calvo-Garrido J. Maffezzini C. Schober F. A. Clemente P. Uhlin E. Kele M. (2019). SQSTM1/p62-directed metabolic reprogramming is essential for normal neurodifferentiation. *Stem Cell Rep.* 12 696–711. 10.1016/j.stemcr.2019.01.023 30827875 PMC6449840

[B25] Caner H. Atasever A. Kilinç K. Durgun B. Peker S. Ozean O. E. (1993). kcta N urochirurgica lipid peroxide level increase in experimental Hydrocephalus. *Acta Neurochir.* 121 68–71. 10.1007/BF01405185 8475810

[B26] Casares-Crespo L. Calatayud-Baselga I. García-Corzo L. Mira H. (2018). On the role of Basal autophagy in adult neural stem cells and neurogenesis. *Front. Cell. Neurosci.* 12:339. 10.3389/fncel.2018.00339 30349462 PMC6187079

[B27] Chandel N. S. (2021). Mitochondria. *Cold Spring Harb. Perspect. Biol.* 13:a040543. 10.1101/cshperspect.a040543 33649187 PMC7919390

[B28] Chandel N. S. Jasper H. Ho T. T. Passegué E. (2016). Metabolic regulation of stem cell function in tissue homeostasis and organismal ageing. *Nat. Cell Biol.* 18 823–832. 10.1038/ncb3385 27428307

[B29] Chen J. Zacharek A. Li A. Cui X. Roberts C. Lu M. (2008). Atorvastatin promotes presenilin-1 expression and Notch1 activity and increases neural progenitor cell proliferation after stroke. *Stroke* 39 220–226. 10.1161/STROKEAHA.107.490946 18063826 PMC2792764

[B30] Chen K. Lu P. Beeraka N. M. Sukocheva O. A. Madhunapantula S. V. Liu J. (2020). Seminars in Cancer Biology Mitochondrial mutations and mitoepigenetics?: Focus on regulation of oxidative stress-induced responses in breast cancers. *Semin. Cancer Biol.* 83 556–569. 10.1016/j.semcancer.2020.09.012 33035656

[B31] Chorna N. E. Santos-Soto I. J. Carballeira N. M. Morales J. L. de La Nuez J. Cátala-Valentin A. (2013). Fatty acid synthase as a factor required for exercise-induced cognitive enhancement and dentate gyrus cellular proliferation. *PLoS One* 8:e77845. 10.1371/journal.pone.0077845 24223732 PMC3818398

[B32] Cliff T. S. Dalton S. (2017). Metabolic switching and cell fate decisions: Implications for pluripotency, reprogramming and development. *Curr. Opin. Genet. Dev.* 30 44–49. 10.1016/j.gde.2017.06.008 28662447 PMC5842063

[B33] Dantzer R. O’Connor J. C. Freund G. G. Johnson R. W. Kelley K. W. (2008). From inflammation to sickness and depression: When the immune system subjugates the brain. *Nat. Rev. Neurosci.* 9 46–56. 10.1038/nrn2297 18073775 PMC2919277

[B34] del Bigio M. R. (1995). The ependyma: A protective barrier between brain and cerebrospinal fluid. *Glia* 14 1–13. 10.1002/glia.440140102 7615341

[B35] del Bigio M. R. (2010). Ependymal cells: Biology and pathology. *Acta Neuropathol.* 119 55–73. 10.1007/s00401-009-0624-y 20024659

[B36] Delgehyr N. Meunier A. Faucourt M. Grau M. B. Strehl L. Janke C. (2015). Ependymal cell differentiation, from monociliated to multiciliated cells. *Methods Cell Biol.* 127 19–35. 10.1016/bs.mcb.2015.01.004 25837384

[B37] Deng Z. Zhou X. Lu J. H. Yue Z. (2021). Autophagy deficiency in neurodevelopmental disorders. *Cell Biosci.* 11:214. 10.1186/s13578-021-00726-x 34920755 PMC8684077

[B38] Du W. Jiang P. Mancuso A. Stonestrom A. Brewer M. D. Minn A. J. (2013). TAp73 enhances the pentose phosphate pathway and supports cell proliferation. *Nat. Cell Biol.* 15 991–1000. 10.1038/ncb2789 23811687 PMC3733810

[B39] Duan X. Kang E. Liu C. Y. Ming G. L. Song H. (2008). Development of neural stem cell in the adult brain. *Curr. Opin. Neurobiol.* 18 108–115. 10.1016/j.conb.2008.04.001 18514504 PMC2464621

[B40] Etschmaier K. Becker T. Eichmann T. O. Schweinzer C. Scholler M. Tam-Amersdorfer C. (2011). Adipose triglyceride lipase affects triacylglycerol metabolism at brain barriers. *J. Neurochem.* 119 1016–1028. 10.1111/j.1471-4159.2011.07498.x 21951135

[B41] Fawal M. A. Davy A. (2018). Impact of Metabolic Pathways and Epigenetics on Neural Stem Cells. *Epigenetics Insights* 11. 10.1177/2516865718820946 30627699 PMC6311566

[B42] Fishman K. Baure J. Zou Y. Huang T. T. Andres-Mach M. Rola R. (2009). Radiation-induced reductions in neurogenesis are ameliorated in mice deficient in CuZnSOD or MnSOD. *Free Radic. Biol. Med.* 47 1459–1467. 10.1016/j.freeradbiomed.2009.08.016 19703553 PMC2767469

[B43] Gijs H. L. Willemarck N. Vanderhoydonc F. Khan N. A. Dehairs J. Derua R. (2015). Primary cilium suppression by SREBP1c involves distortion of vesicular trajficking by PLA2G3. *Mol. Biol. Cell* 26 2321–2332. 10.1091/mbc.E14-10-1472 25904332 PMC4462948

[B44] Go A. (2012). Somatic progenitor cell vulnerability to Mitochondrial DNA Mutagenesis underlies progeroid phenotypes in polg mutator mice. *Cell Metab.* 15 100–109. 10.1016/j.cmet.2011.11.012 22225879

[B45] Götz M. Huttner W. B. (2005). The cell biology of neurogenesis. *Nat. Rev. Mol. Cell Biol.* 6 777–788. 10.1038/nrm1739 16314867

[B46] Grarup N. Moltke I. Andersen M. K. Dalby M. Vitting-Seerup K. Kern T. (2018). Loss-of-function variants in ADCY3 increase risk of obesity and type 2 diabetes. *Nat. Genet.* 50 172–174. 10.1038/s41588-017-0022-7 29311636 PMC5828106

[B47] Haack T. B. Ignatius E. Calvo-garrido J. Iuso A. Suomalainen A. Gorza M. (2016). Absence of the autophagy adaptor SQSTM1/p62 causes childhood-onset neurodegeneration with ataxia, dystonia, and gaze palsy. *Am. J. Hum. Genet.* 99 735–743. 10.1016/j.ajhg.2016.06.026 27545679 PMC5010644

[B48] Hamilton L. K. Dufresne M. Joppé S. E. Petryszyn S. Aumont A. Calon F. (2015). Aberrant lipid metabolism in the forebrain niche suppresses adult neural stem cell proliferation in an Animal Model of Alzheimer’s Disease. *Cell Stem Cell* 17 397–411. 10.1016/j.stem.2015.08.001 26321199

[B49] Hampshire D. J. Ayub M. Springell K. Roberts E. Jafri H. Rashid Y. (2006). MORM syndrome (mental retardation, truncal obesity, retinal dystrophy and micropenis), a new autosomal recessive disorder, links to 9q34. *Eur. J. Hum. Genet.* 14 543–548. 10.1038/sj.ejhg.5201577 16493448

[B50] Han Y. M. Kang G. M. Byun K. Ko H. W. Kim J. Shin M. S. (2014). Leptin-promoted cilia assembly is critical for normal energy balance. *J. Clin. Investig.* 124 2193–2197. 10.1172/JCI69395 24667636 PMC4001529

[B51] He Z. Liu H. Agostini M. Yousefi S. Perren A. Tschan M. P. (2013). P73 regulates autophagy and hepatocellular lipid metabolism through a transcriptional activation of the ATG5 gene. *Cell Death Differ.* 20 1415–1424. 10.1038/cdd.2013.104 23912709 PMC3770317

[B52] Higginbotham H. Guo J. Yokota Y. Umberger N. L. Su C. Y. Li J. (2013). Arl13b-regulated cilia activities are essential for polarized radial glial scaffold formation. *Nat. Neurosci.* 16 1000–1007. 10.1038/nn.3451 23817546 PMC3866024

[B53] Hirano K. Namihira M. (2016). LSD1 mediates neuronal differentiation of human fetal neural stem cells by controlling the expression of a novel target gene. HEYL. *Stem Cells* 34 1872–1882. 10.1002/stem.2362 27018646

[B54] Hisahara S. Chiba S. Matsumoto H. Tanno M. Yagi H. Shimohama S. (2008). Histone deacetylase SIRT1 modulates neuronal differentiation by its nuclear translocation. *Proc. Natl. Acad. Sci. U.S.A.* 105 15599–15604. 10.1073/pnas.0800612105 18829436 PMC2563076

[B55] Homem C. C. F. Steinmann V. Burkard T. R. Jais A. Esterbauer H. Knoblich J. A. (2014). Ecdysone and mediator change energy metabolism to terminate proliferation in *Drosophila* neural stem cells. *Cell* 158 874–888. 10.1016/j.cell.2014.06.024 25126791

[B56] Hu B. Guo Y. Chen C. Li Q. Niu X. Guo S. (2014). Repression of SIRT1 promotes the differentiation of mouse induced pluripotent stem cells into neural stem cells. *Cell. Mol. Neurobiol.* 34 905–912. 10.1007/s10571-014-0071-8 24832395 PMC11488914

[B57] Hunter M. Demarais N. J. Faull R. L. M. Grey A. C. Curtis M. A. (2018). Subventricular zone lipidomic architecture loss in Huntington’s disease. *J. Neurochem.* 146 613–630. 10.1111/jnc.14468 29804301

[B58] Imai S. I. Guarente L. (2014). NAD^+^ and sirtuins in aging and disease. *Trends Cell Biol.* 24 464–471. 10.1016/j.tcb.2014.04.002 24786309 PMC4112140

[B59] Irmak D. Fatima A. Gutiérrez-Garcia R. Rinschen M. M. Wagle P. Altmüller J. (2018). Mechanism suppressing H3K9 trimethylation in pluripotent stem cells and its demise by polyQ-expanded huntingtin mutations. *Hum. Mol. Genet.* 27 4117–4134. 10.1093/hmg/ddy304 30452683

[B60] Ito K. Suda T. (2014). Metabolic requirements for the maintenance of self-renewing stem cells. *Nat. Rev. Mol. Cell Biol.* 15 243–256. 10.1038/nrm3772 24651542 PMC4095859

[B61] Iwata R. Casimir P. Vanderhaeghen P. (2020). Mitochondrial dynamics in postmitotic cells regulate neurogenesis. *Science* 369 858–862. 10.1126/science.aba9760 32792401

[B62] Jackson P. K. Attardi L. D. (2016). P73 and FoxJ1: Programming multiciliated epithelia. *Trends Cell Biol.* 26 239–240. 10.1016/j.tcb.2016.03.001 26988441 PMC5555749

[B63] Jain A. Lamark T. Sjøttem E. Larsen K. B. Awuh J. A. Øvervatn A. (2010). p62/SQSTM1 Is a target gene for transcription factor NRF2 and creates a positive feedback loop by inducing antioxidant response element-driven gene transcription. *J. Biol. Chem.* 285 22576–22591. 10.1074/jbc.M110.118976 20452972 PMC2903417

[B64] Jang J. Wang Y. Lalli M. A. Guzman E. Godshalk S. E. Zhou H. (2016). Primary cilium-autophagy-Nrf2 (PAN) axis activation commits human embryonic stem cells to a Neuroectoderm Fate. *Cell* 165 410–420. 10.1016/j.cell.2016.02.014 27020754

[B65] Kacher R. Lamazière A. Heck N. Kappes V. Mounier C. Despres G. (2019). CYP46A1 gene therapy deciphers the role of brain cholesterol metabolism in Huntington’s disease. *Brain* 142 2432–2450. 10.1093/brain/awz174 31286142

[B66] Kempermann G. Song H. Gage F. H. (2015). Neurogenesis in the adult hippocampus. *Cold Spring Harb. Perspect. Med.* 7:a018812. 10.1101/cshperspect.a018812 26330519 PMC4563705

[B67] Khacho M. Slack R. S. (2018). Mitochondrial dynamics in the regulation of neurogenesis?: From development to the Adult Brain. *Dev. Dyn.* 247 47–53. 10.1002/dvdy.24538 28643345

[B68] Khacho M. Clark A. Svoboda D. S. Harper M. E. Park D. S. Slack R. S. (2016). Mitochondrial dynamics impacts stem cell identity and fate decisions by regulating a nuclear transcriptional program. *Stem Cell* 19 232–247. 10.1016/j.stem.2016.04.015 27237737

[B69] Khacho M. Clark A. Svoboda D. S. Maclaurin J. G. Lagace D. C. Park D. S. (2017). Mitochondrial dysfunction underlies cognitive defects as a result of neural stem cell depletion and impaired neurogenesis. *Hum. Mol. Genet.* 26 3327–3341. 10.1093/hmg/ddx217 28595361 PMC5886206

[B70] Khatri P. Obernier K. Simeonova I. K. Hellwig A. Hölzl-Wenig G. Mandl C. (2014). Proliferation and cilia dynamics in neural stem cells prospectively isolated from the SEZ. *Sci. Rep.* 4:3803. 10.1038/srep03803 24448162 PMC3898048

[B71] Kishimoto N. Sawamoto K. (2012). Planar polarity of ependymal cilia. *Differentiation* 83:S86–S90. 10.1016/j.diff.2011.10.007 22101065

[B72] Knobloch M. (2017). The role of lipid metabolism for neural stem cell regulation. *Brain Plast.* 3 61–71. 10.3233/BPL-160035 29765860 PMC5928532

[B73] Knobloch M. Jessberger S. (2017). ScienceDirect metabolism and neurogenesis. *Curr. Opin. Neurobiol.* 42 45–52. 10.1016/j.conb.2016.11.006 27915086

[B74] Knobloch M. Braun S. M. G. Zurkirchen L. von Schoultz C. Zamboni N. Araúzo-Bravo M. J. (2013). Metabolic control of adult neural stem cell activity by Fasn-dependent lipogenesis. *Nature* 493 226–230. 10.1038/nature11689 23201681 PMC3587167

[B75] Knobloch M. Pilz G. A. Ghesquière B. Kovacs W. J. Wegleiter T. Moore D. L. (2017). A fatty acid oxidation-dependent metabolic shift regulates adult neural stem cell activity. *Cell Rep.* 20 2144–2155. 10.1016/j.celrep.2017.08.029 28854364 PMC5583518

[B76] Kokoeva M. V. Yin H. Flier J. S. (2005). Neurogenesis in the Hypothalamus of Adult mice: Potential role in energy balance. *Science* 310 679–683. 10.1126/science.1115360 16254185

[B77] Komatsu M. Kurokawa H. Waguri S. Taguchi K. Kobayashi A. Sou Y. S. (2010). The selective autophagy substrate p62 activates the stress responsive transcription factor Nrf2 through inactivation of Keap1. *Nat. Cell Biol.* 12 213–223. 10.1038/ncb2021 20173742

[B78] Kondziella D. Sonnewald U. Tullberg M. Wikkelso C. (2008). Brain metabolism in adult chronic hydrocephalus. *J. Neurochem.* 106 1515–1524. 10.1111/j.1471-4159.2008.05422.x 18419769

[B79] Kopinke D. Roberson E. C. Reiter J. F. (2017). Ciliary hedgehog signaling restricts injury-induced adipogenesis. *Cell* 170 340–351.e12. 10.1016/j.cell.2017.06.035 28709001 PMC5617351

[B80] Krashes M. J. Lowell B. B. Garfield A. S. (2016). Melanocortin-4 receptor-regulated energy homeostasis. *Nat. Neurosci.* 19 206–219. 10.1038/nn.4202 26814590 PMC5244821

[B81] Kyrousi C. Arbi M. Pilz G. A. Pefani D. E. Lalioti M. E. Ninkovic J. (2015). Mcidas and gemc1 are key regulators for the generation of multiciliated ependymal cells in the adult neurogenic niche. *Development* 142 3661–3674. 10.1242/dev.126342 26395491

[B82] Kyrousi C. Lygerou Z. Taraviras S. (2017). How a radial glial cell decides to become a multiciliated ependymal cell. *Glia* 65 1032–1042. 10.1002/glia.23118 28168763

[B83] Lalioti M. E. Kaplani K. Lokka G. Georgomanolis T. Kyrousi C. Dong W. (2019b). GemC1 is a critical switch for neural stem cell generation in the postnatal brain. *Glia* 67 2360–2373. 10.1002/glia.23690 31328313

[B84] Lalioti M. E. Arbi M. Loukas I. Kaplani K. Kalogeropoulou A. Lokka G. (2019a). GemC1 governs multiciliogenesis through direct interaction with and transcriptional regulation of p73. *J. Cell Sci.* 132:jcs228684. 10.1242/jcs.228684 31028178

[B85] Le Belle J. E. Orozco N. M. Paucar A. A. Saxe J. P. Mottahedeh J. Pyle A. D. (2011). Proliferative neural stem cells have high endogenous ROS levels that regulate self-renewal and neurogenesis in a PI3K/Akt-dependant manner. *Stem Cell* 8 59–71. 10.1016/j.stem.2010.11.028 21211782 PMC3018289

[B86] Lee C. H. Song D. K. Park C. B. Choi J. Kang G. M. Shin S. H. (2020). Primary cilia mediate early life programming of adiposity through lysosomal regulation in the developing mouse hypothalamus. *Nat. Commun.* 11:5772. 10.1038/s41467-020-19638-4 33188191 PMC7666216

[B87] Lee C. Hu J. Ralls S. Kitamura T. Loh Y. P. Yang Y. (2012). The Molecular profiles of neural stem cell niche in the Adult Subventricular Zone. *PLoS One* 7:e50501. 10.1371/journal.pone.0050501 23209762 PMC3510163

[B88] Lee K. M. Hwang S. K. Lee J. A. (2013). Neuronal autophagy and neurodevelopmental disorders. *Exp. Neurobiol.* 22 133–142. 10.5607/en.2013.22.3.133 24167408 PMC3807000

[B89] Li G. Jiapaer Z. Weng R. Hui Y. Jia W. Xi J. (2017). Dysregulation of the SIRT1/OCT6 Axis contributes to environmental stress-induced neural induction defects. *Stem Cell Rep.* 8 1270–1286. 10.1016/j.stemcr.2017.03.017 28434941 PMC5425630

[B90] Lim D. A. Tramontin A. D. Trevejo J. M. Herrera D. G. Manuel García-Verdugo J. Alvarez-Buylla A. (2000). Noggin antagonizes BMP signaling to create a niche for Adult neurogenesis. *Neuron* 28 713–726. 10.1016/S0896-6273(00)00148-3 11163261

[B91] Llorens-bobadilla E. Zhao S. Baser A. Saiz-castro G. Martin-villalba A. Llorens-bobadilla E. (2015). Single-cell transcriptomics reveals a population of dormant neural stem cells that become activated upon Brain Injury. *Stem Cell* 17 329–340. 10.1016/j.stem.2015.07.002 26235341

[B92] Lu A. T. Narayan P. Grant M. J. Langfelder P. Wang N. Kwak S. (2020). DNA methylation study of Huntington’s disease and motor progression in patients and in animal models. *Nat. Commun.* 11:4529. 10.1038/s41467-020-18255-5 32913184 PMC7484780

[B93] Lummis N. C. Sánchez-Pavón P. Kennedy G. Frantz A. J. Kihara Y. Blaho V. A. (2019). LPA 1/3 overactivation induces neonatal posthemorrhagic hydrocephalus through ependymal loss and ciliary dysfunction. *Sci. Adv.* 5:eaax2011. 10.1126/sciadv.aax2011 31633020 PMC6785248

[B94] Ma C. Y. Yao M. J. Zhai Q. W. Jiao J. W. Yuan X. B. Poo M. M. (2014). SIRT1 suppresses self-renewal of adult hippocampal neural stem cells. *Development* 141 4697–4709. 10.1242/dev.117937 25468938

[B95] Ma H. Folmes C. D. L. Wu J. Morey R. Mora-castilla S. Ocampo A. (2015). Metabolic rescue in pluripotent cells from patients with mtDNA disease. *Nature* 524 234–238. 10.1038/nature14546 26176921

[B96] Maerz L. D. Burkhalter M. D. Schilpp C. Wittekindt O. H. Frick M. Philipp M. (2019). Pharmacological cholesterol depletion disturbs ciliogenesis and ciliary function in developing zebrafish. *Commun. Biol.* 2:31. 10.1038/s42003-018-0272-7 30729178 PMC6351647

[B97] Maffezzini C. Calvo-Garrido J. Wredenberg A. Freyer C. (2020). Metabolic regulation of neurodifferentiation in the Adult Brain. *Cell. Mol. Life Sci.* 77 2483–2496. 10.1007/s00018-019-03430-9 31912194 PMC7320050

[B98] Maharjan Y. Lee J. N. Kwak S. A. Dutta R. K. Park C. Choe S. (2020). TMEM135 regulates primary ciliogenesis through modulation of intracellular cholesterol distribution. *EMBO Rep.* 21:e48901. 10.15252/embr.201948901 32157776 PMC7202201

[B99] Malatesta P. Appolloni I. Calzolari F. (2008). Radial glia and neural stem cells. *Cell and Tissue Res.* 331 165–178. 10.1007/s00441-007-0481-8 17846796

[B100] Marin R. Rojo J. A. Fabelo N. Fernandez C. E. Diaz M. (2013). Lipid raft disarrangement as a result of neuropathological progresses: A novel strategy for early diagnosis? *Neuroscience* 245 26–39. 10.1016/j.neuroscience.2013.04.025 23618758

[B101] Marion V. Stoetzel C. Schlicht D. Messaddeq N. Koch M. Flori E. (2008). *Proc. Natl. Acad. Sci. U.S.A.* 106 1820–1825. 10.1073/pnas.0812518106 19190184 PMC2635307

[B102] Martínez-reyes I. Chandel N. S. (2020). Mitochondrial TCA cycle metabolites control. *Nat. Commun.* 11:102. 10.1038/s41467-019-13668-3 31900386 PMC6941980

[B103] Maryanovich M. Gross A. (2013). A ROS rheostat for cell fate regulation. *Trends Cell Biol.* 23 129–134. 10.1016/j.tcb.2012.09.007 23117019

[B104] McDonnell E. Crown S. B. Fox D. B. Kitir B. Ilkayeva O. R. Olsen C. A. (2016). Lipids reprogram metabolism to become a major carbon source for histone acetylation. *Cell Rep.* 17 1463–1472. 10.1016/j.celrep.2016.10.012 27806287 PMC5123807

[B105] Merkle F. T. Zaman M. Arturo A. B. (2007). Mosaic organization of neural stem cells in the Adult Brain. *Science* 317 381–384. 10.1126/science.1144914 17615304

[B106] Miceli C. Roccio F. Penalva-Mousset L. Burtin M. Leroy C. Nemazanyy I. (2020). The primary cilium and lipophagy translate mechanical forces to direct metabolic adaptation of kidney epithelial cells. *Nat. Cell Biol.* 22 1091–1102. 10.1038/s41556-020-0566-0 32868900

[B107] Milošević N. J. Judaš M. Aronica E. Kostovic I. (2014). Neural ECM in laminar organization and connectivity development in healthy and diseased human brain. *Prog. Brain Res.* 214 159–178. 10.1016/B978-0-444-63486-3.00007-4 25410357

[B108] Ming G. L. Song H. (2005). Adult neurogenesis in the *Mammalian* central nervous system. *Annu. Rev. Neurosci.* 28 223–250. 10.1146/annurev.neuro.28.051804.101459 16022595

[B109] Mira H. Morante J. (2020). Neurogenesis from embryo to adult – lessons from flies and mice. *Front. Cell Dev. Biol.* 8:533. 10.3389/fcell.2020.00533 32695783 PMC7339912

[B110] Mirzadeh Z. Han Y. G. Soriano-Navarro M. García-Verdugo J. M. Alvarez-Buylla A. (2010). Cilia organize ependymal planar polarity. *J. Neurosci.* 30 2600–2610. 10.1523/JNEUROSCI.3744-09.2010 20164345 PMC2873868

[B111] Mirzadeh Z. Merkle F. T. Soriano-Navarro M. Garcia-Verdugo J. M. Alvarez-Buylla A. (2008). Neural stem cells confer unique pinwheel architecture to the ventricular surface in neurogenic regions of the Adult Brain. *Cell Stem Cell* 3 265–278. 10.1016/j.stem.2008.07.004 18786414 PMC2613692

[B112] Molnár Z. Clowry G. J. Šestan N. Alzu’bi A. Bakken T. Hevner R. F. (2019). New insights into the development of the human cerebral cortex. *J. Anat.* 235 432–451. 10.1111/joa.13055 31373394 PMC6704245

[B113] Murphy A. R. Laslett A. O’Brien C. M. Cameron N. R. (2017). Scaffolds for 3D *in vitro* culture of neural lineage cells. *Acta Biomater.* 54 1–20. 10.1016/j.actbio.2017.02.046 28259835

[B114] Muto V. Flex E. Kupchinsky Z. Primiano G. (2018). Biallelic SQSTM1 mutations in early-onset, variably progressive neurodegeneration. *Neurology* 91:e319–e330. 10.1212/WNL.0000000000005869 29959261 PMC6070386

[B115] Namba T. Dóczi J. Pinson A. Xing L. Kalebic N. Wilsch-Bräuninger M. (2020). Human-specific ARHGAP11B Acts in mitochondria to expand neocortical progenitors by glutaminolysis. *Neuron* 105 867–881.e9. 10.1016/j.neuron.2019.11.027 31883789

[B116] Namba T. Nardelli J. Gressens P. Huttner W. B. (2021). Metabolic regulation of neocortical expansion in development and evolution. *Neuron* 109 408–419. 10.1016/j.neuron.2020.11.014 33306962

[B117] Nemajerova A. Amelio I. Gebel J. Dötsch V. Melino G. Moll U. M. (2018). Non-oncogenic roles of TAp73: From multiciliogenesis to metabolism. *Cell Death Differ.* 25 144–153. 10.1038/cdd.2017.178 29077094 PMC5729534

[B118] Niklison-Chirou M. V. Agostini M. Amelio I. Melino G. (2020). Regulation of adult neurogenesis in *Mammalian* brain. *Int. J. Mol. Sci.* 21:4869. 10.3390/ijms21144869 32660154 PMC7402357

[B119] O’Brien L. C. Keeney P. M. Bennett J. P. (2015). Differentiation of human neural stem cells into motor neurons stimulates mitochondrial biogenesis and decreases Glycolytic Flux. *Stem Cells Dev.* 24 1984–1994. 10.1089/scd.2015.0076 25892363 PMC4545371

[B120] Obernier K. Alvarez-Buylla A. (2019). Neural stem cells: Origin, heterogeneity and regulation in the adult *Mammalian* brain. *Development* 146:dev156059. 10.1242/dev.156059 30777863 PMC6398449

[B121] Oh E. C. Vasanth S. Katsanis N. (2015). Metabolic regulation and energy homeostasis through the primary cilium. *Cell Metab.* 21 21–31. 10.1016/j.cmet.2014.11.019 25543293 PMC4370781

[B122] Okun E. Marton D. Cohen D. Griffioen K. Kanfi Y. Illouz T. (2017). Sirt6 alters adult hippocampal neurogenesis. *PLoS One* 12:e0179681. 10.1371/journal.pone.0179681 28644902 PMC5482455

[B123] Ortiz-Álvarez G. Daclin M. Shihavuddin A. Lansade P. Fortoul A. Faucourt M. (2019). Adult neural stem cells and multiciliated ependymal cells share a common lineage regulated by the geminin family members. *Neuron* 102 159–172.e7. 10.1016/j.neuron.2019.01.051 30824354 PMC6449116

[B124] Ou Y. Ruan Y. Cheng M. Moser J. J. Rattner J. B. van der Hoorn F. A. (2009). Adenylate cyclase regulates elongation of *Mammalian* primary cilia. *Exp. Cell Res.* 315 2802–2817. 10.1016/j.yexcr.2009.06.028 19576885 PMC3161028

[B125] Paez-Gonzalez P. Abdi K. Luciano D. Liu Y. Soriano-Navarro M. Rawlins E. (2011). Ank3-dependent SVZ Niche assembly is required for the continued production of new neurons. *Neuron* 71 61–75. 10.1016/j.neuron.2011.05.029 21745638 PMC3134799

[B126] Rafalski V. A. Ho P. P. Brett J. O. Ucar D. Dugas J. C. Pollina E. A. (2013). Expansion of oligodendrocyte progenitor cells following SIRT1 inactivation in the adult brain. *Nat. Cell Biol.* 15 614–624. 10.1038/ncb2735 23644469 PMC4026158

[B127] Renault V. M. Rafalski V. A. Morgan A. A. Salih D. A. M. Brett J. O. Webb A. E. (2009). FoxO3 regulates neural stem cell homeostasis. *Cell Stem Cell* 5 527–539. 10.1016/j.stem.2009.09.014 19896443 PMC2775802

[B128] Ringers C. Olstad E. W. Jurisch-Yaksi N. (2020). The role of motile cilia in the development and physiology of the nervous system. *Philos. Trans. R. Soc. B* 375:20190156. 10.1098/rstb.2019.0156 31884916 PMC7017337

[B129] Rodgers J. T. Lerin C. Haas W. Gygi S. P. Spiegelman B. M. Puigserver P. (2005). Nutrient control of glucose homeostasis through a complex of PGC-1α and SIRT1. *Nature* 434 113–118. 10.1038/nature03354 15744310

[B130] Rodrigues D. C. Harvey E. M. Suraj R. Erickson S. L. Mohammad L. Ren M. (2020). Methylglyoxal couples metabolic and translational control of notch signalling in *Mammalian* neural stem cells. *Nat. Commun.* 11:2018. 10.1038/s41467-020-15941-2 32332750 PMC7181744

[B131] Rojczyk-Gołębiewska E. Pałasz A. Wiaderkiewicz R. (2014). Hypothalamic subependymal niche: A novel site of the adult neurogenesis. *Cell. Mol. Neurobiol.* 34 631–642. 10.1007/s10571-014-0058-5 24744125 PMC4047487

[B132] Romani P. Valcarcel-Jimenez L. Frezza C. Dupont S. (2021). Crosstalk between mechanotransduction and metabolism. *Nat. Rev. Mol. Cell Biol.* 22 22–38. 10.1038/s41580-020-00306-w 33188273

[B133] Sabbisetti V. di Napoli A. Seeley A. Amato A. M. O’Regan E. Ghebremichael M. (2009). p63 promotes cell survival through fatty acid synthase. *PLoS One* 4:e5877. 10.1371/journal.pone.0005877 19517019 PMC2691576

[B134] Saharan S. Jhaveri D. J. Bartlett P. F. (2013). SIRT1 regulates the neurogenic potential of neural precursors in the adult subventricular zone and hippocampus. *J. Neurosci. Res.* 91 642–659. 10.1002/jnr.23199 23404532

[B135] Satir P. Christensen S. T. (2007). Overview of structure and function of *Mammalian* cilia. *Annu. Rev. Physiol.* 69 377–400. 10.1146/annurev.physiol.69.040705.141236 17009929

[B136] Shabani Z. Ghadiri T. Karimipour M. Sadigh-Eteghad S. Mahmoudi J. Mehrad H. (2021). Modulatory properties of extracellular matrix glycosaminoglycans and proteoglycans on neural stem cells behavior: Highlights on regenerative potential and bioactivity. *Int. J. Biol. Macromol.* 171 366–381. 10.1016/j.ijbiomac.2021.01.006 33422514

[B137] Sheng X. Sheng Y. Gao S. Fan F. Wang J. (2020). Low fluid shear stress promoted ciliogenesis via Dvl2 in hUVECs. *Histochem. Cell Biol.* 154 639–654. 10.1007/s00418-020-01908-3 32776193

[B138] Shimabukuro M. K. Langhi L. G. P. Cordeiro I. Brito J. M. Batista C. M. D. C. Mattson M. P. (2016). Lipid-laden cells differentially distributed in the aging brain are functionally active and correspond to distinct phenotypes. *Sci. Rep.* 6:23795. 10.1038/srep23795 27029648 PMC4814830

[B139] Shyh-Chang N. Ng H. H. (2017). The metabolic programming of stem cells. *Genes Dev.* 31 336–346. 10.1101/gad.293167.116 28314766 PMC5358754

[B140] Siyahhan B. Knobloch V. de Zélicourt D. Asgari M. Daners M. S. Poulikakos D. (2014). Flow induced by ependymal cilia dominates near-wall cerebrospinal fluid dynamics in the lateral ventricles. *J. R. Soc. Interface* 11:20131189. 10.1098/rsif.2013.1189 24621815 PMC3973363

[B141] Solozobova V. Wyvekens N. Pruszak J. (2012). Lessons from the embryonic neural stem cell niche for neural lineage differentiation of pluripotent stem cells. *Stem Cell Rev. Rep.* 8 813–829. 10.1007/s12015-012-9381-8 22628111 PMC3412081

[B142] Steib K. Schäffner I. Jagasia R. Ebert B. Chichung Lie D. (2014). Mitochondria modify exercise-induced development of stem cell-derived neurons in the adult brain. *J. Neurosci.* 34 6624–6633. 10.1523/JNEUROSCI.4972-13.2014 24806687 PMC6608139

[B143] Stein L. R. Imai S. I. (2012). The dynamic regulation of NAD metabolism in mitochondria. *Trends Endocrinol. Metab.* 23 420–428. 10.1016/j.tem.2012.06.005 22819213 PMC3683958

[B144] Stein L. R. Imai S. I. (2014). Specific ablation of Nampt in adult neural stem cells recapitulates their functional defects during aging. *EMBO J.* 33 1321–1340. 10.1002/embj.201386917 24811750 PMC4194122

[B145] Stoll E. A. Makin R. Sweet I. R. Trevelyan A. J. Miwa S. Horner P. J. (2015). Neural stem cells in the adult subventricular zone oxidize fatty acids to produce energy and support neurogenic activity. *Stem Cells* 33 2306–2319. 10.1002/stem.2042 25919237 PMC4478223

[B146] Su X. Gi Y. J. Chakravarti D. Chan I. L. Zhang A. Xia X. (2012). TAp63 is a master transcriptional regulator of lipid and glucose metabolism. *Cell Metab.* 16 511–525. 10.1016/j.cmet.2012.09.006 23040072 PMC3483083

[B147] Takahashi K. Nagai T. Chiba S. Nakayama K. Mizuno K. (2018). Glucose deprivation induces primary cilium formation through mTORC1 inactivation. *J. Cell Sci.* 131:jcs208769. 10.1242/jcs.208769 29180513

[B148] Tavazoie M. van der Veken L. Silva-Vargas V. Louissaint M. Colonna L. Zaidi B. (2008). A specialized vascular niche for adult neural stem cells. *Cell Stem Cell* 3 279–288. 10.1016/j.stem.2008.07.025 18786415 PMC6864413

[B149] Taverna E. Götz M. Huttner W. B. (2014). The cell biology of neurogenesis: Toward an understanding of the development and evolution of the neocortex. *Annu. Rev. Cell Dev. Biol.* 30 465–502. 10.1146/annurev-cellbio-101011-155801 25000993

[B150] Tong C. K. Han Y. G. Shah J. K. Obernier K. Guinto C. D. Alvarez-Buylla A. (2014). Primary cilia are required in a unique subpopulation of neural progenitors. *Proc. Natl. Acad. Sci. U.S.A.* 111 12438–12443. 10.1073/pnas.1321425111 25114218 PMC4151724

[B151] Tsatmali M. Walcott E. C. Crossin K. L. (2005). Newborn neurons acquire high levels of reactive oxygen species and increased mitochondrial proteins upon differentiation from progenitors. *Brain Res.* 1040 137–150. 10.1016/j.brainres.2005.01.087 15804435

[B152] van Noorden C. J. F. Breznik B. Novak M. van Dijck A. J. Tanan S. Vittori M. (2022). Cell biology meets cell metabolism: Energy production is similar in stem cells and in Cancer stem cells in brain and bone marrow. *J. Histochem. Cytochem.* 79 29–51. 10.1369/00221554211054585 34714696 PMC8721571

[B153] Vázquez P. Arroba A. I. Cecconi F. de La Rosa E. J. Boya P. de Pablo F. (2012). Atg5 and Ambra1 differentially modulate neurogenesis in neural stem cells. *Autophagy* 8 187–199. 10.4161/auto.8.2.18535 22240590

[B154] Walton N. M. Shin R. Tajinda K. Heusner C. L. Kogan J. H. Miyake S. (2012). Adult neurogenesis transiently generates oxidative stress. *PLoS One* 7:e35264. 10.1371/journal.pone.0035264 22558133 PMC3340368

[B155] Wang C. Chen S. Yeo S. Uzunbas G. K. White E. Mizushima N. (2016). Elevated p62/SQSTM1 determines the fate of autophagy-deficient neural stem cells by increasing superoxide. *J. Cell Biol.* 212 545–560. 10.1083/jcb.201507023 26929451 PMC4772497

[B156] Wang C. Liang C. C. Bian Z. C. Zhu Y. Guan J. L. (2013). FIP200 is required for maintenance and differentiation of postnatal neural stem cells. *Nat. Neurosci.* 16 532–542. 10.1038/nn.3365 23542691 PMC3637881

[B157] Webb A. E. Pollina E. A. Vierbuchen T. Urbán N. Ucar D. Leeman D. S. (2013). FOXO3 shares common targets with ASCL1 genome-wide and inhibits ASCL1-dependent neurogenesis. *Cell Rep.* 4 477–491. 10.1016/j.celrep.2013.06.035 23891001 PMC3838667

[B158] Xie Z. Jones A. Deeney J. T. Hur S. K. Bankaitis V. A. (2016). Inborn errors of long-chain fatty acid β-Oxidation link neural stem cell self-renewal to autism. *Cell Rep.* 14 991–999. 10.1016/j.celrep.2016.01.004 26832401 PMC4749429

[B159] Yamada S. Ishikawa M. Nozaki K. (2021). Exploring mechanisms of ventricular enlargement in idiopathic normal pressure hydrocephalus: A role of cerebrospinal fluid dynamics and motile cilia. *Fluids Barriers CNS* 18:20. 10.1186/s12987-021-00243-6 33874972 PMC8056523

[B160] Yamakawa D. Katoh D. Kasahara K. Shiromizu T. Matsuyama M. Matsuda C. (2021). Primary cilia-dependent lipid raft/caveolin dynamics regulate adipogenesis. *Cell Rep.* 34:108817. 10.1016/j.celrep.2021.108817 33691104

[B161] Yambire K. F. Oliveira P. J. Raimundo N. Signaling O. (2020). Mitochondria – lysosome crosstalk?: From physiology to neurodegeneration. *Trends Mol. Med.* 26 71–88. 10.1016/j.molmed.2019.10.009 31791731

[B162] Yao B. Christian K. M. He C. Jin P. Ming G. L. Song H. (2016). Epigenetic mechanisms in neurogenesis. *Nat. Rev. Neurosci.* 17 537–549. 10.1038/nrn.2016.70 27334043 PMC5610421

[B163] Yildirim F. Ng C. W. Kappes V. Ehrenberger T. Rigby S. K. Stivanello V. (2019). Early epigenomic and transcriptional changes reveal Elk-1 transcription factor as a therapeutic target in Huntington’s disease. *Proc. Natl. Acad. Sci. U.S.A.* 116 24840–24851. 10.1073/pnas.1908113116 31744868 PMC6900520

[B164] Yousef H. Morgenthaler A. Schlesinger C. Bugaj L. Conboy I. M. Schaffer D. V. (2015). Age-associated increase in BMP signaling inhibits hippocampal neurogenesis. *Stem Cells* 33 1577–1588. 10.1002/stem.1943 25538007 PMC4818000

[B165] Zhang H. Ryu D. Wu Y. Gariani K. Wang X. Luan P. (2016). Supplementary Materials for enhances life span in mice. *Science* 352 1436–1443. 10.1126/science.aaf2693 27127236

[B166] Zhang Z. Gao F. Kang X. Li J. Zhang L. Dong W. (2015). Exploring the potential relationship between Notch pathway genes expression and their promoter methylation in mice hippocampal neurogenesis. *Brain Res. Bull.* 113 8–16. 10.1016/j.brainresbull.2015.02.003 25701255

[B167] Zheng X. Boyer L. Jin M. Mertens J. Kim Y. Ma L. (2016). Metabolic reprogramming during neuronal differentiation from aerobic glycolysis to neuronal oxidative phosphorylation. *Elife* 5:e13374. 10.7554/eLife.13374 27282387 PMC4963198

[B168] Zhou W. Zhao T. Du J. Ji G. Li X. Ji S. (2019). TIGAR promotes neural stem cell differentiation through acetyl-CoA-mediated histone acetylation. *Cell Death Dis.* 10:198. 10.1038/s41419-019-1434-3 30814486 PMC6393469

[B169] Zhu Q. Mariash A. Margosian M. R. Gopinath S. Fareed M. T. Anderson G. W. (2001). Spot 14 gene deletion increases hepatic de novo lipogenesis. *Endocrinology* 142 4363–4370. 10.1210/endo.142.10.8431 11564699

